# Mending a broken heart with novel cardiogenic small molecules

**DOI:** 10.1186/s13619-022-00120-z

**Published:** 2022-05-05

**Authors:** Nevan Powers, Guo N. Huang

**Affiliations:** 1 Cardiovascular Research Institute & Department of Physiology, University of California, San Francisco, San Francisco, CA 94158 USA; 2 Eli and Edythe Broad Center for Regeneration Medicine and Stem Cell Research, University of California, San Francisco, San Francisco, CA 94158 USA

## Abstract

Adult mammalian cardiomyocytes are unable to proliferate to regenerate lost tissue after heart injury. Du et al., reporting in *Cell Stem Cell*, employ a FUCCI- and MADM-based system to screen for small molecules combinations that produced a collaborative effect on cardiomyocyte cycling and cytokinesis. The authors generate a cocktail of five small molecules that increase cardiomyocyte proliferation and regeneration in vitro and in vivo with high efficiency, and explore its potential in cardiac regenerative repair after myocardial infarction through a new potential pathway for cardiomyocyte cell-cycle re-entry.

## Main text

**Fig. 1:**
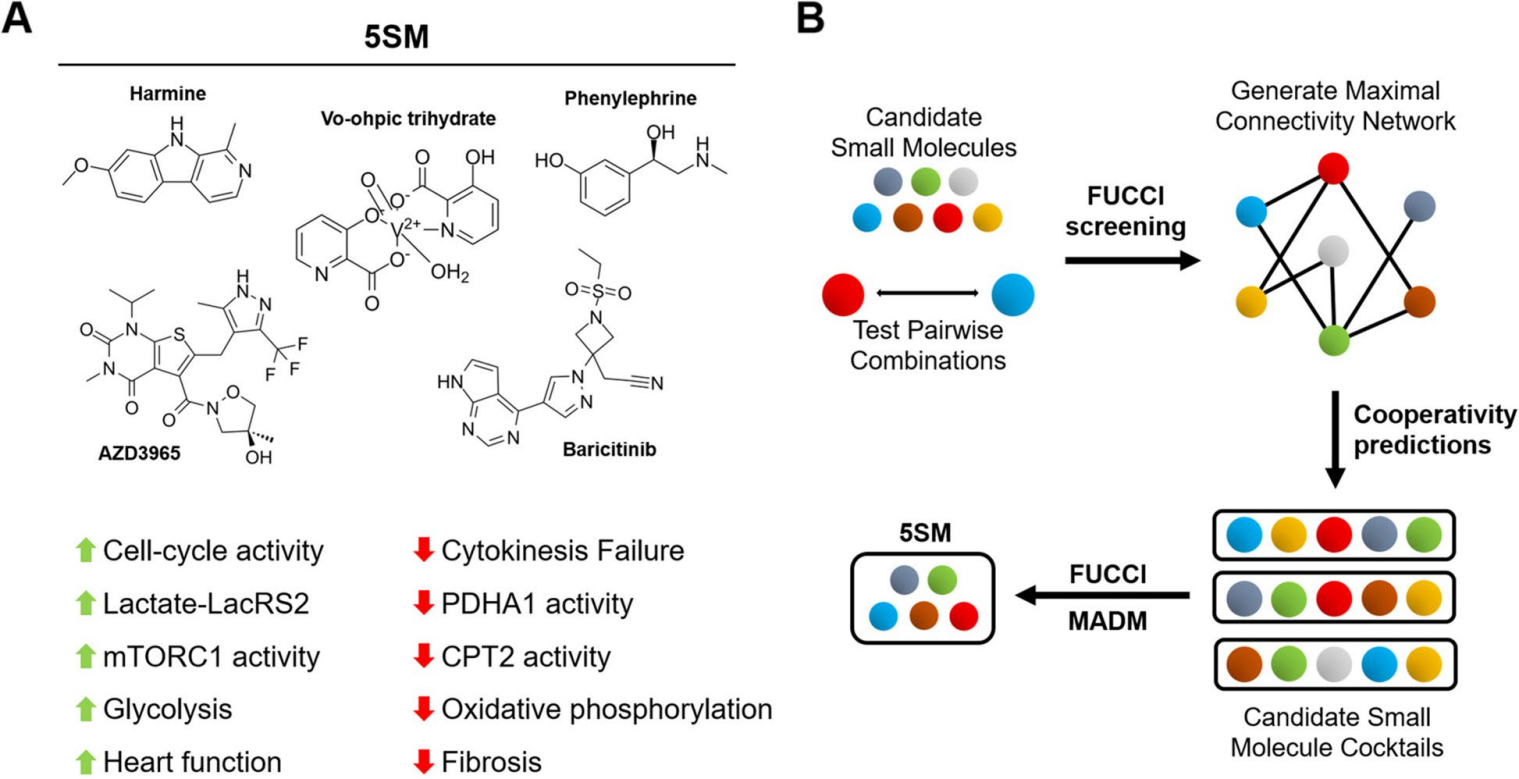
Discovery of a five small molecule cocktail (5SM) that promotes cardiomyocyte proliferation and heart regeneration in adult mammals. (**A**) Small molecule components of 5SM and their combined effects on cardiomyocyte functions. (**B**) Schematic of the small molecule combination screening process to discover 5SM

Heart disease is the leading cause of death across the globe, owing to the inability of the adult human heart to functionally regenerate after injury. This lack of regenerative ability is thought to be shared by the majority of adult mammals, as mammalian cardiomyocytes undergo cell cycle exit during development and lose their ability to proliferate to replace any lost or damaged tissue following injury (Garbern & Lee, 2022; Khyeam et al., 2021; Cutie & Huang, 2021). Genetic tracing experiments in animals with high regenerative capacity of the heart, such as adult zebrafish and neonatal mice, provide compelling evidence to support that new cardiomyocytes originate from preexisting cardiomyocytes through rapid proliferation (Garbern & Lee, 2022; Khyeam et al., 2021; Cutie & Huang, 2021). These studies have inspired intense investigations on why adult mammalian cardiomyocytes only retain limited proliferative potential and how to induce expansion of preexisting cardiomyocytes for cardiac repair (Garbern & Lee, 2022). Small molecules that induce cardiomyocyte proliferation are excellent candidates to achieve this purpose. Indeed, chemical compounds such as regulators of Hippo, GSK and Wnt pathways are able to promote cardiomyocyte cell-cycle re-entry and produce a regenerative effect in the adult mammalian heart (Hara et al., 2018; Woulfe et al., 2010; Fan et al., 2020). While previous iterations of these chemicals were often limited by the degree of their effectiveness, Du et al. (Du et al., 2022) show the discovery of a cocktail of five small molecules that produces robust proliferation in cardiomyocytes of different sources, including adult human primary cardiomyocytes, and promotes impressive heart functional improvement in adult rats after injury. They further explore the mechanisms behind this induction of proliferation to unveil the involvement of a LacRS2-mediated signaling pathway previously unappreciated in cardiac regeneration (Fig. 1A).

Using a Fluorescent Ubiquination-based Cell Cycle Indicator (FUCCI)- based system to report cell cycle activity in isolated neonatal rat cardiomyocytes, Du et al. were able to identify 13 candidate small molecules by screening 564 small molecules by their effect on cardiomyocyte proliferation. Next, they tested the pairwise effects of these 13 candidate small molecules on cardiomyocyte cell-cycle re-entry. They developed a maximal connectivity algorithm to create interactive networks based on the collaborative effects of small molecule pairs to identify small molecules with high connectivity and synergy, resulting in the identification of ten small molecule combinations that were predicted to have the strongest interactions. These ten cocktails were tested against each other in the same FUCCI system to assess cell cycle re-entry into S/G2/M phases, but also through a Mosaic Analysis with Double Markers (MADM)- based system to identify successful cytokinesis (Fig. 1B). Through their assay, Du et al. identified the small molecule combination that produced the strongest positive effect on cardiomyocyte cell-cycle re-entry and cytokinesis: phenylephrine, baricitinib, harmine, vo-ohpic trihydrate, and AZD3965. These small molecules are known to target α1-adrenergic receptor, JAK1, DYRK, PTEN, and MCT1, respectively. While the initial screening was performed using neonatal rat and mouse cardiomyocytes that are known to already have innate proliferative capacity, this combination of small molecules was also shown to have a robust pro-proliferative effect on hiPSC-derived and adult human atrial cardiomyocytes that are largely non-proliferative in vitro.

After identifying and demonstrating the effectiveness of the optimal five small molecule cocktail (5SM) in vitro, Du et al. explored the effectiveness of 5SM on cardiomyocyte proliferation and regeneration in vivo. They used immunostaining to show that 5SM increased cardiomyocyte proliferation after intramyocardial injection in adult rat hearts, and examined a MADM mouse line to observe the increase in cardiomyocyte cytokinesis after 5SM treatment in vivo. Additionally, Du et al. found that 5SM largely increased the percentage of cardiomyocytes that expressed cell-cycle markers within the injury border regions after induction of myocardial infarction in adult rat hearts. They further examined effects of 5SM on cardiac regeneration over the course of 8 weeks after injury through fibrotic staining and echocardiography, finding that 5SM greatly reduced the final fibrotic area and improved cardiac performance throughout the recovery period. Impressively, 5SM treatment increased the function of injured hearts to levels comparable to that of the sham injury, indicating successful functional regeneration of the heart. 5SM appeared to increase cardiac function through non-hypertrophic methods, as cardiomyocyte cross-sectional area was comparable after injury in both treated and non-treated groups.

With a robust regenerative effect in vivo, the mechanisms of exactly how 5SM induced cardiomyocyte proliferation required further investigation. Du et al. performed gene expression and chromatin landscape analysis on neonatal rat cardiomyocytes after 5SM treatment. They observed that 5SM-treated cardiomyocytes showed an upregulation of many cell division-related processes such as DNA replication and chromosome separation through increased gene expression as well as chromatin accessibility, as expected from the observed increase in proliferation of treated cardiomyocytes. Interestingly, the 5SM treatment had no effect on well-studied cardiomyocyte proliferation pathways Hippo and pERK (Garbern & Lee, 2022). Treatment also increased gene expression within the PI3K-AKT, glycolysis, and dedifferentiation pathways while decreasing expression related to oxidative phosphorylation and differentiation. Upregulation of dedifferentiation markers was confirmed using single cell RNA-seq, suggesting that 5SM promoted cardiomyocyte proliferation by inducing the dedifferentiation of quiescent cardiomyocytes before cell division. Sequencing results also suggested that 5SM induced a dramatic metabolic change in affected cardiomyocytes, resulting in the shift from oxidative phosphorylation to glycolysis. These results were confirmed through direct measurement of the lactate concentrations within 5SM-treated cardiomyocytes. The number of FUCCI-positive cells could be elevated by directly increasing lactate concentration in culture medium, which was though to inhibit mitochondrial metabolism through increased LacRS2-mediated lactylation of PDHA1 and CPT2. Subsequent accumulation of anabolic metabolites was expected to increase mTORC1 activity and biosynthetic pathways enabling the activation of processes essential for cell-cycle re-entry. Du et al. observed that depletion of LacRS2 via siRNA or direct inhibition by β-alanine, as well as mTORC1 siRNA knockdown eliminated the pro-proliferative effect of 5SM. Together, their results suggested that 5SM induced cardiomyocyte proliferation by increasing intracellular lactate concentrations, resulting in the overall metabolic switch from oxidative phosphorylation to glycolysis and thus activating the cell-cycle re-entry processes downstream of mTORC1.

## Conclusions

Du et al. discovered a cocktail of five small molecules that induces cardiomyocyte proliferation and striking functional improvement in vivo following myocardial infarction in adult rats. Their cocktail shows clinical promise in its ability to induce proliferation in primary human atrial cardiomyocytes and hiPSC-derived cardiomyocytes in vitro, and with its small molecule platform enabling easier translatability than other transgene or viral treatments that require genetic modification. In exploring the mechanism of how 5SM induces cardiomyocyte proliferation, Du et al. revealed a previously unknown lactate-induced mechanism for quiescent cardiomyocyte cell-cycle re-entry through LacRS2-mediate metabolic signaling changes. This new mechanism for small molecule-induced dedifferentiation and cardiomyocyte cell-cycle re-entry opens the door for research into pathways that may drive highly efficient adult mammalian cardiomyocyte proliferation and regeneration. In addition to these novel advancements, their study also raises several questions: Are all five small molecules necessary to promote heart regeneration? Do these chemicals induce new vasculature formation and modulate activation of non-cardiomyocyte populations such as immune cells and fibroblasts to support regenerative repair? Can this cocktail also improve heart function of larger mammals after ischemic injury? These exciting steps for further explanation will build upon the findings of Du et al. to progress towards the goal of regenerating the human heart.

## Data Availability

Not applicable.
